# miR-511-3p dysregulation-mediated AKT3/USP8 signaling imbalance: a molecular bridge between neuroinflammation and PSCI

**DOI:** 10.3389/fimmu.2026.1766326

**Published:** 2026-04-28

**Authors:** Wei Zhao, Kangping Song, Fen Yang, Hui Xiao, Yan Liu, Yachun Yu, Te Wang

**Affiliations:** 1 Department of Neurology, The Affiliated Changsha Central Hospital, Hengyang Medical School, University of South China, Changsha, Hunan, China; 2 Department of Science and Education, The Affiliated Changsha Central Hospital, Hengyang Medical School, University of South China, Changsha, Hunan, China

**Keywords:** AKT3, inflammatory responses, miR-511-3p, neuroinflammation, post-stroke cognitive impairment, stroke, USP8

## Abstract

**Background:**

This study aims to explore the regulatory effect of miR-511-3p expression imbalance on the AKT3/USP8 signaling pathway, as well as the “molecular bridge” function of this signaling imbalance between neuroinflammation and post-stroke cognitive impairment (PSCI).

**Methods:**

118 stroke patients and 80 healthy individuals were enrolled. Bioinformatics and dual-luciferase assays confirmed miR-511-3p - AKT3 interaction. Oxygen-glucose deprivation/reoxygenation (OGD/R) established a stroke inflammatory cell model. Inflammatory cytokines were measured via ELISA. RT-qPCR detected miR-511-3p, AKT3, and USP8 expressions. Correlation analysis and risk factor analysis were performed using Pearson correlation and logistic regression.

**Results:**

This study found that AKT3 was a direct target of miR-511-3p. In the neural inflammation cell model, miR-511-3p could regulate the expression of AKT3/USP8; miR-511-3p/AKT3/USP8 could regulate the inflammatory response in the inflammatory cell model. In the serum of PSCI patients, the expression of miR-511-3p and USP8 were decreased, while the expressions of AKT3 was increased, and all three were associated with the inflammatory factors of the patients. Logistic regression analysis showed that low expression of miR-511-3p and USP8 were independent risk factors for PSCI.

**Conclusion:**

miR-511-3p may regulate the expression of USP8 by targeting AKT3, thereby influencing the neuroinflammatory response and participating in the occurrence and development of PSCI. miR-511-3p and USP8 may potentially serve as biomarkers and therapeutic targets for PSCI.

## Introduction

Stroke is an acute neurologic injury caused by ischemia or hemorrhage that stems from a wide range of pathologies (1). Post-stroke cognitive impairment (PSCI) is a common long-term complication for stroke patients. Approximately one-third of stroke patients will develop PSCI, which significantly affects their quality of life and even their survival time (2). The inflammatory process in stroke involves activating various cells, including microglia, astrocytes, endothelial cells, and leukocytes. It also causes the release of pro-inflammatory cytokines such as inflammatory chemokines (e.g., TNF-α, IL-1β and IL-6) and adhesion molecules (3, 4). Persistent neuroinflammation can disrupt the neuronal connections in brain regions related to cognition such as the hippocampus, inhibit neurogenesis, and ultimately lead to a decline in cognitive function (5). However, there is a lack of a clear molecular pathway connecting “neuroinflammation” with “PSCI”.

microRNA (miRNA) is a class of 20–25 nucleotides of non-coding RNA, which can regulate the transcription and translation of target genes by binding to the 3’UTR region of the mRNA of target genes (6). Previous studies have shown that miR-511-3p plays an important role in a variety of diseases, including tumors, cardiovascular diseases and nervous system diseases. For example, miR-511-3p participates in the development of AD by regulating the levels of neuroinflammatory factors in AD cells (7). In MCAO model, downregulation of miR-511-3p is associated with neurological dysfunction and aggravated inflammation (8). Studies have found that miR-511-3p targets AKT3 and inhibits its activity, thereby affecting cell proliferation, apoptosis and migration and other biological functions (9, 10), this direct targeting relationship of miR-511-3p to AKT3 has also been confirmed in prostate cancer, where their abnormal expression is associated with poor prognosis (11). AKT3 is one of the core members of the PI3K/AKT signaling pathway, which plays an important role in neuroinflammation. For example, miR-122-5p promotes inflammation in the brain by inhibiting MLLT1/PI3K/AKT signaling pathway (12); miR-124 reduces cerebral I/R injury by negatively regulating the PI3K/AKT/mTOR pathway (13). AKT3 activation may promote the de-ubiquitination of USP8, which affects the activation of NF-κB (14, 15). Emerging evidence suggests that ubiquitin-specific protease 8 (USP8), a key deubiquitinating enzyme, plays an important regulatory role in neuroinflammation and neurological injury (16–18). Recent studies have demonstrated that USP8 can modulate microglial activation and inflammatory signaling pathways, including the NF-κB and NLRP3 inflammasome pathways, thereby influencing the release of pro-inflammatory cytokines and neuronal injury after ischemic stroke (19). Therefore, USP8 was selected as a potential downstream regulatory molecule involved in the miR-511-3p/AKT3 signaling cascade in the present study. Although the regulatory role of miR-511-3p in inflammation and the role of the AKT3/USP8 signaling pathway in neural function have been preliminarily reported, the association between the two and their role in “neuroinflammation - PSCI” have not yet been studied.

This study aims to explore the role of the expression change of miR-511-3p in PSCI, and to determine whether it regulates the neuroinflammatory response after stroke by mediating the imbalance of the AKT3/USP8 signaling pathway; ultimately to verify the scientific hypothesis that “miR-511-3p-AKT3/USP8 signaling imbalance” serves as a “molecular bridge between neuroinflammation and PSCI”, providing new theoretical basis for the mechanism research and targeted intervention of PSCI.

## Materials and methods

### Research subjects

This study enrolled 118 stroke patients and 80 healthy controls from the The Affiliated Changsha Central Hospital, Hengyang Medical School, University of South China. Following were the inclusion criteria: (1) patients were diagnosed according to the diagnostic criteria for acute stroke; (2) the clinical records of acute stroke patients were completed. The cases with a history of stroke, severe heart diseases, severe infection diseases, dissection, liver and nephrosis disease, or any malignant tumors were excluded from our study. This cohort study estimated sample size based on the two-sample t-test formula (α=0.05, 1-β=0.8, medium effect size) and the EPV rule (≥10 events per variable) for logistic regression. A total of 118 stroke patients and 80 healthy controls were enrolled, meeting the statistical power requirements. Venous blood was collected from the participants, centrifuged and stored at -80°C for subsequent application. The protocols of this study were approved by the Ethics Committee of the The Affiliated Changsha Central Hospital, Hengyang Medical School, University of South China, and written informed consent was obtained from each participant.

### Evaluation of cognitive function

Cognitive function was assessed using the Montreal Cognitive Assessment (MoCA) at admission and 6 months after stroke diagnosis (the key time point for diagnosis). According to the MoCA score (cut-off value: 22 points), stroke patients were divided into post-stroke cognitive impairment (PSCI, MoCA ≤22) and post-stroke cognitive normal (PSCN, MoCA >22) groups. The specific operational procedures of the MoCA assessment were strictly carried out in accordance with the standardized scheme described in reference (20) to ensure the consistency of the assessment process.

### Cell culture and treatment

The mouse microglia cell line BV2 cells (Procell, China, CL-0697) were resuscitated, seeded in six-well plates, added with DMEM containing 10% fetal bovine serum (FBS). And cells cultured in a constant temperature incubator at 37°C and 5% CO2 for 24 hours. The cells in logarithmic phase cells were used for experiments.

BV2 cells were subjected to OGD/R experiment 24 hours after inoculation. The cell medium was removed and BV2 cells were washed with glucose-free DMEM for several times. Then BV2 cells were cultured in glucose-free DMEM in a hypoxic incubator (1% O_2_, 94% N_2_, 5% CO_2_) for 6 h. After that, the glucose-free DMEM was removed, and BV2 cells were grown in complete medium for 24 h. The culture conditions were simulated the reperfusion. BV2 cells in the control group were treated identically except that they were not exposed to OGD.

The OGD/R-stimulated BV2 cells model was then treated with transfection according to experimental requirements. The miR-511-3p mimic, mimic NC were synthesized by RIBOBIO (Guangzhou, China). The AKT3, USP8 was subcloned into the vector pcDNA3.1 (Invitrogen, Carlsbad, CA, USA), generating the vector pcDNA3.1-AKT3, pcDNA3.1-USP8. The negative control vector pcDNA3.1-NC was used as a control. Plasmids and miR-511-3p mimic/mimic NC at a final concentration of 50 nM were transfected into the cells using Lipofectamine 2000 (Invitrogen) following the manufacturer’s protocol. Transfected cells were incubated at 37°C for 48 h and then were available for the following experiments.

### Dual-luciferase reporter assay

We used the starBase database (https://starbase.sysu.edu.cn/) to predict binding sites. The sequences of AKT3 containing miR-511-3p binding sites were subcloned into dual luciferase reporter plasmids (Promega, USA) to construct wild-type recombinant luciferase reporter plasmids AKT3-WT, and the mutation sequences of the binding sites were inserted into the plasmids to construct mutant luciferase reporter plasmids AKT3-MUT. BV2 cells were inoculated into 24-well plates, and the above recombinant plasmids were mixed with miR-511-3p mimic, mimic NC and miR-511-3p inhibitor, inhibitor NC, and transfected under Lipofectamine 3000. After 48 hours, the relative luciferase activity was evaluated by dual-luciferase reporter assay.

### Evaluation of inflammatory responses

The inflammatory responses were evaluated by measuring the levels of pro-inflammatory cytokines. The concentration of IL-1β (cat#29-8012-65, Thermo Fisher Scientific, USA), IL-6 (cat#29-8061-65, Thermo Fisher Scientific, USA) and TNF-α (cat#29-831-65, Thermo Fisher Scientific, USA) in serum and cell supernatants were measured according to the instruction of the commercially available ELISA kits. 100 μL of the sample was added to the ELISA plates and wash after incubated with the antibody for 60 min. 100 μL HRP working solution was added and incubated for 30 min. The substrate was added and incubated in the dark for 15 min, and finally the termination solution was supplemented. Absorbance at 450 nm was determined using a Multiskan microplate reader.

### Reverse transcription-quantitative polymerase chain reaction

Total RNA was extracted by RNA Extraction Reagent (Invitrogen, Shanghai, China). And the quality of Total RNA was evaluated using NanoDrop 2000 spectrophotometer (Thermo Fisher Scientific, USA). The RNA then was reversely transcribed into cDNA using a reverse transcription Kit (TaKaRa, Kusatsu, Japan). qPCR was conducted using SYBR green I Master Mix kit (Invitrogen, Carlsbad, CA, USA) and a real-time fluorescent qPCR 7500 system (Applied Biosystems, USA). The primer sequences used in the reactions were synthesized by Sangon (Shanghai, China) and listed in **Table 1**. AKT3 and USP8 were normalized to intracellular GAPDH, and miR-511-3p was normalized to U6. The relative expression levels of miR-511-3p, AKT3 and USP8 were calculated by the 2^−ΔΔCT^ method.

**Table 1:** Nucleotide fragments and primer sequences used in study.

Gene	Sequences (5’-3’)	
miR-511-3p AKT3 USP8	Forward	GCAGAATGTGTAGCAAAAGACA
	Reverse	GGTCCAGTTTTTTTTTTTTTTTATCCT
	Forward	TGTGGATTTACCTTATCCCCTCA
	Reverse	GTTTGGCTTTGGTCGTTCTGT
	Forward	AGACTCTCCGAAAGCCTTAAACT
	Reverse	GCCGTTAATCCTTTGGGTTTTGG
GAPDH U6	Forward	AGGTCGGTGTGAACGGATTTG
	Reverse	TGTAGACCATGTAGTTGAGGTCA
	Forward	GCTTCGGCAGCACATATACTAAAAT
	Reverse	CGCTTCACGAATTTGCGTGTCAT

### Statistical analysis

SPSS 27.0 statistical software (IBM Corp., Armonk, NY, USA), GraphPad Prism 9.0 (GraphPad Software, San Diego, CA, USA) and R software (version 4.2.1, R Foundation for Statistical Computing, Vienna, Austria) were adopted for statistical analyses. For the baseline clinical and demographic characteristics of the study population, one-way analysis of variance (ANOVA) was applied for continuous variables, and the Chi-square (χ²) test was used for categorical variables. Independent sample *t*-tests was used to analyze the differences in gene expression between the two groups, and one-way ANOVA was adopted to compare the differences between multiple groups. Pearson correlation coefficient was used to evaluate the correlation between miR-511-3p, AKT3, USP8 and inflammatory cytokines expression in neuroinflammation. Using Logistic regression analysis, the correlations between miR-511-3p, AKT3 and USP8 and the occurrence of PSCI were investigated. Multivariate logistic regression was adjusted for age, gender, smoking status, drinking status, diabetes and hypertension (all covariates were assessed via clinical medical records and patient questionnaires). Receiver operating characteristic (ROC) curves were constructed to evaluate the diagnostic performance of miR-511-3p and USP8 in distinguishing PSCI from PSCN. The area under the curve (AUC), cutoff value, sensitivity, specificity, calibration curve, Hosmer−Lemeshow test, and bootstrap validation accuracy were further calculated. The results were considered significant at *P* < 0.05.

## Results

### AKT3 was a direct target of miR-511-3p

**Figure 1:**
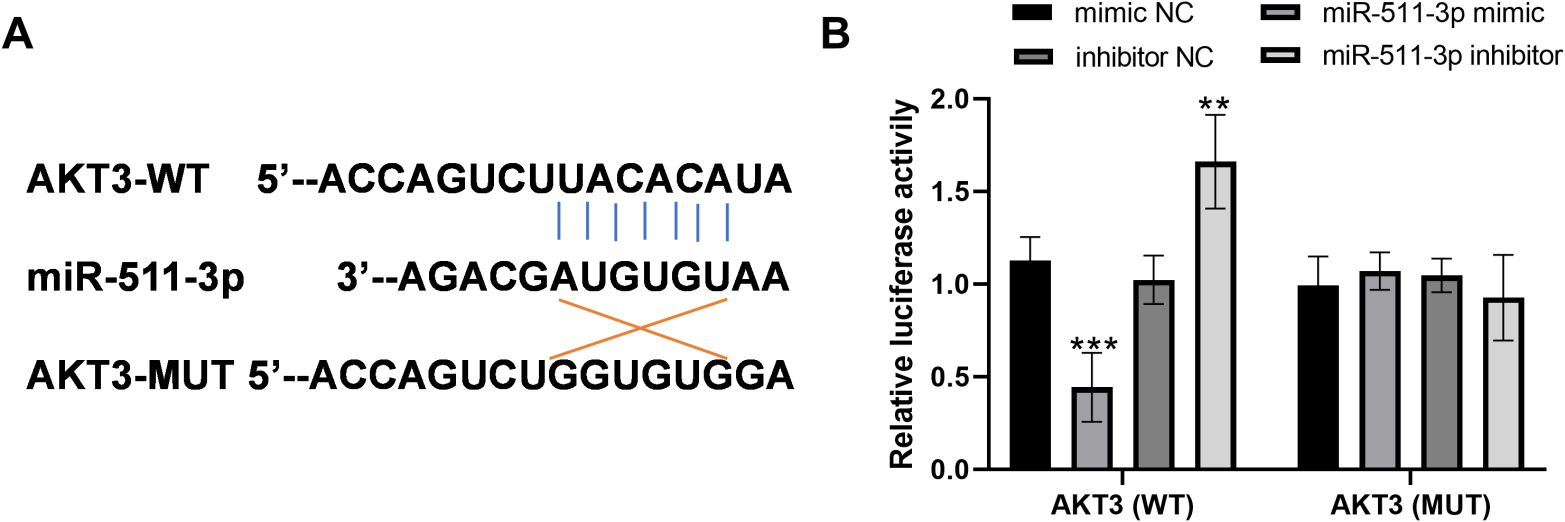
AKT3 was a direct target of miR-511-3p. **(A)** Complementary sequences of miR-511-3p in the 3’UTR of AKT3. **(B)** Results of dual-luciferase reporter assay. ** *P* < 0.01, *** *P* < 0.001, compared to mimic NC.

The sequence of the 3’-UTR of AKT3, which contained the complementary sequences of miR-511-3p, were predicted according to the starBase (**Figure 1A**). The following dual-luciferase reporter assay showed that miR-511-3p overexpression inhibited the relative luciferase activity, while the reduction of miR-511-3p performed reversed function in the wild-type group in BV2 cells (**Figure 1B**, all *P<0.01*). However, miR-511-3p mimic or inhibitor transfection did not affect luciferase activity in cells transfected with AKT3-MUT (**Figure 1B**).

### Regulatory effects of miR-511-3p on the AKT3/USP8 in BV2 cell model

**Figure 2:**
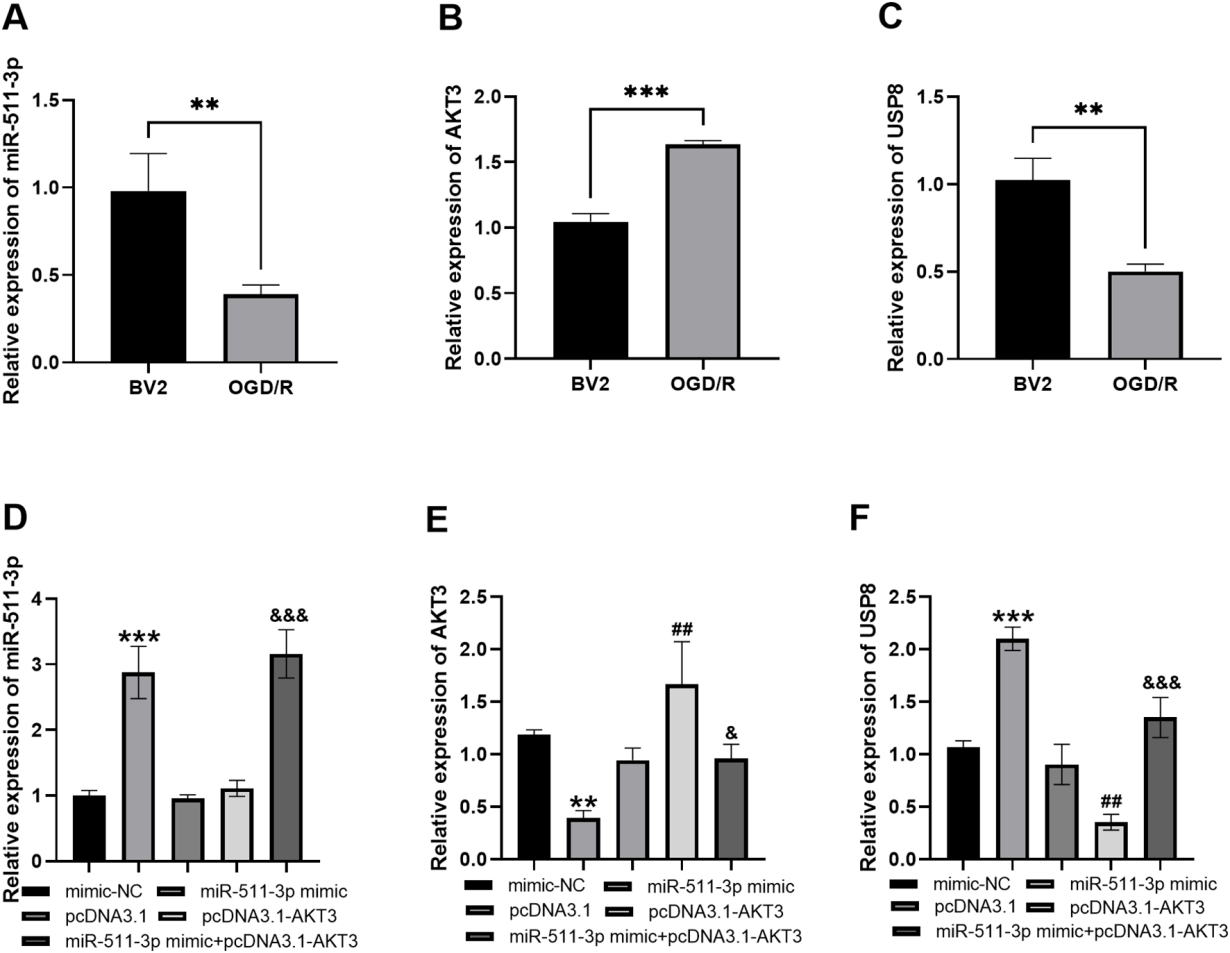
Regulatory effects of miR-511-3p on the AKT3/USP8 in BV2 cells. **(A–C)**. Relative expression of miR-511-3p/AKT3/USP8 in BV2 cell model. **(D)**. miR-511-3p mimic promoted miR-511-3p expression. **(E)**. pcDNA3.1-AKT3 promoted AKT3 expression, but miR-511-3p mimic inhibited AKT3 expression, while this inhibition was rescued by pcDNA3.1-AKT3. **(F)**. pcDNA3.1-AKT3 inhibited USP8 expression, but miR-511-3p mimic promoted USP8 expression, while this promotion was rescued by pcDNA3.1-AKT3. ** *P* < 0.01, *** *P* < 0.001, compared to mimic NC; ## *P* < 0.01, compared to pcDNA3.1; & *P* < 0.05, &&& *P* < 0.001, compared to miR-511-3p mimic.

By using OGD/R, a neuroinflammatory cell model was established. The expression of miR-511-3p and USP8 was markedly inhibited, but AKT3 expression was significantly promoted in the cell model (**Figures 2A–C**, all *P<0.01*). Transfection efficiency of miR-511-3p mimic was verified by RT-qPCR, which showed a significant upregulation of miR-511-3p expression compared with mimic NC (*P* < 0.001, **Figure 2D**), confirming successful transfection. Besides, miR-511-3p mimic inhibited the expression of AKT3 in the cell model (**Figure 2E**, *P<0.01*), but the inhibited AKT3 was reversed with the co-transfection of pcDNA3.1-AKT3 (**Figure 2E**, *P<0.05*). Furthermore, pcDNA3.1-AKT3 abolished USP8 expression (**Figure 2F**, *P<0.01*), while transfection with miR-511-3p mimic in pcDNA3.1-AKT3 cells significantly restored USP8 levels (**Figure 2F**, *P<0.001*).

### Regulatory effects of miR-511-3p/AKT3/USP8 on inflammatory response in BV2 cells

**Figure 3:**
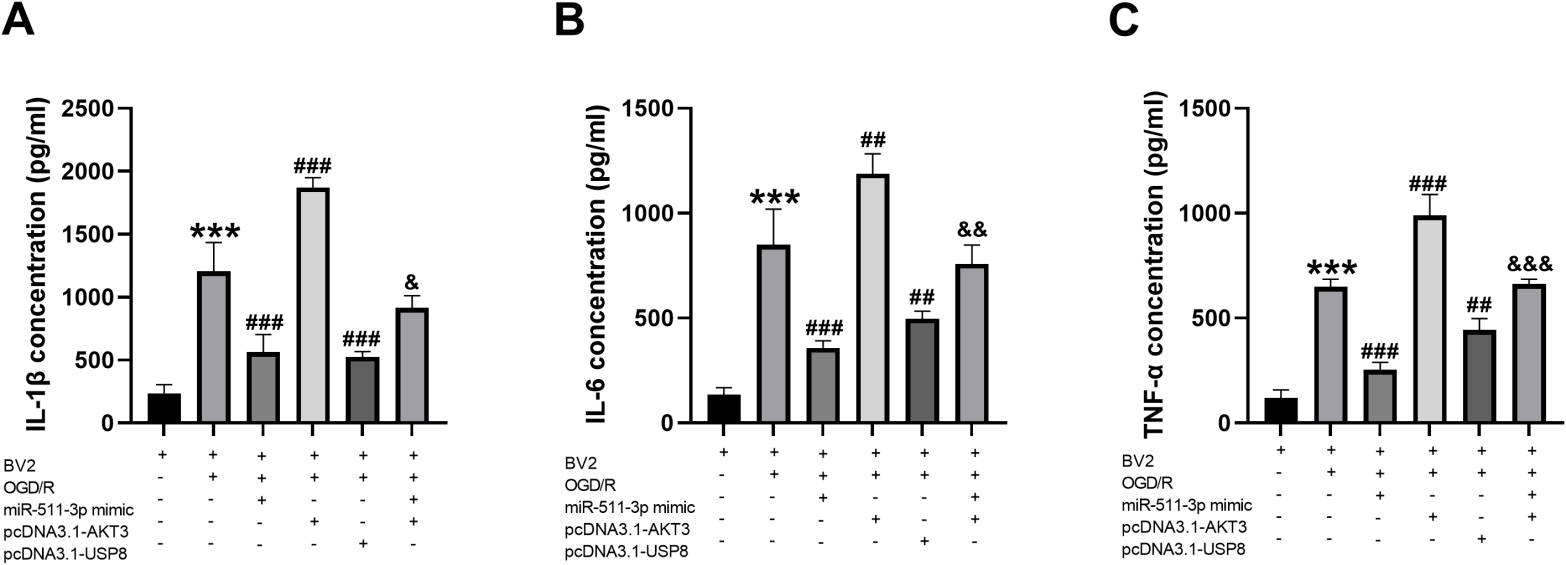
miR-511-3p inhibited inflammation by targeting AKT3/USP8 in the cell model. **(A)** The expression level of IL-1β was regulatory by miR-511-3p/AKT3/USP8. **(B)** The expression level of IL-6 was regulatory by miR-511-3p/AKT3/USP8. **(C)** The expression level of TNF-α was regulatory by miR-511-3p/AKT3/USP8. *** *P* < 0.001, compared to BV2; ## *P* < 0.01, ### *P* < 0.001, compared to BV2+OGD/R; & *P* < 0.05, && *P* < 0.01, &&& *P* < 0.001, compared to BV2+OGD/R+miR-511-3p mimic.

For the inflammatory responses in the cell model, OGD/R-induced significantly increased release of IL-1β, IL-6 and TNF-α (**Figures 3A–C**, all *P<0.001*). The increased inflammatory cytokines were all inhibited when upregulating the expression of miR-511-3p and USP8 (all *P* < 0.05), retrospectively. On the contrary, the overexpression of AKT3 performed promoting effects on the release of inflammatory cytokines (all P < 0.05). Notably, the inhibited inflammation induced by miR-511-3p overexpression was found to be abolished by elevating AKT3 (all *P <0.05*).

### Expression of miR-511-3p, AKT3 and USP8 in the serum samples of PSCI patients

**Figure 4:**
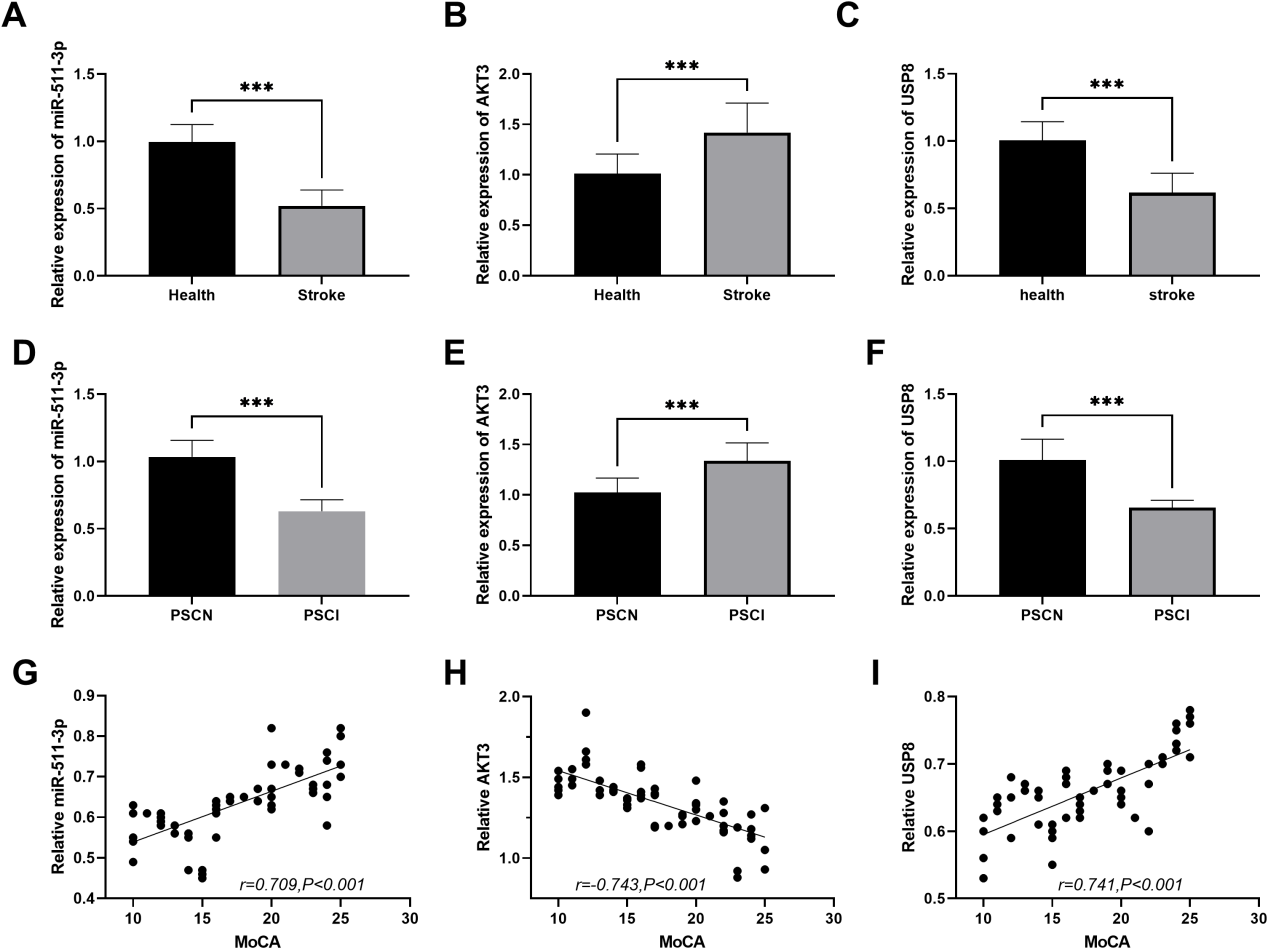
The expression differences of miR-511-3p, AKT3 and USP8 in the serum of healthy individuals and stroke/PSCI patients. A-C. The comparison of expression differences of miR-511-3p, AKT3 and USP8 between the healthy group and the stroke group. D-F. Compared with the PSCN group, the expressions of miR-511-3p and USP8 were decreased in the PSCI group, while the expression of AKT3 was increased. G-I. Evaluation of the correlation between miR-511-3p, AKT3 and USP8 expression and MoCA score. *** *P* < 0.001.

Baseline clinical characteristics analysis (**Table 2**) showed no significant differences in age, BMI, gender, smoking and drinking status among healthy controls, PSCI and PSCN groups (all P>0.05), indicating good baseline comparability between groups; the proportions of diabetes and hypertension in the PSCI group were significantly higher than those in the healthy control group (all *P* < 0.017). **Figure 4A–C** show that, compared with healthy individuals, the relative expression of miR-511-3p and USP8 in stroke patients was significantly decreased, while the relative expression of AKT3 was significantly increased (all *P* < 0.001). **Figures 4D–F** indicate that miR-511-3p, AKT3, and USP8 had the same expression trend in post-stroke cognitive normal (PSCN) and PSCI. Compared with PSCN, the expression of miR-511-3p and USP8 in PSCI patients was decreased, and the expression of AKT3 was increased (all *P* < 0.001). In **Figures 4G–I**, miR-511-3p and USP8 were positively correlated with the MoCA score (r =0.709, *P* < 0.001; r =0.741, *P* < 0.001), while AKT3 was negatively correlated with the MoCA score (r= -0.743, *P* < 0.001), suggesting that these molecules may be related to post-stroke cognitive function.

**Table 2:** Baseline clinical characteristics of the healthy control, PSCN and PSCI groups.

Indicators	Healthy control (n=80)	PSCI (n=66)	PSCN(n=52)	*P* value^*^
Age (years)	57.50 ± 7.16	59.18 ± 7.70	56.60 ± 8.91	0.185
BMI (kg/m^2^)	20.20 ± 2.21	22.29 ± 2.11	22.40 ± 1.93	0.871
Gender, *n*(%)				0.893
Male	40 (50.00)	33 (50.00)	24 (46.15)	
Female	40 (50.00)	33 (50.00)	28 (53.85)	
Smoking, *n*(%)	42 (52.50)	35 (53.03)	26 (50.00)	0.942
Drinking, *n*(%)	43 (53.75)	34 (51.52)	27 (51.92)	0.959
Diabetes, *n*(%)	12 (15.00)	28 (42.42)^a^	15 (28.85)	0.001
Hypertension, *n*(%)	19 (23.75)	33 (50.00)^b^	19 (36.54)	0.004

Values are presented as the means ± SD or n (%).

^*^ p value calculated by ANOVA or the χ² test.

^a^Significant difference (*p* = 0.001<0.017) between PSCI and AMC with the Pearson Chi-Square Test.

^b^Significant difference (*p*<0.001) between PSCI and AMC with the Pearson Chi-Square Test.

### Association of miR-511-3p, AKT3 and USP8 with Inflammatory cytokines in Patients with PSCI

**Figure 5:**
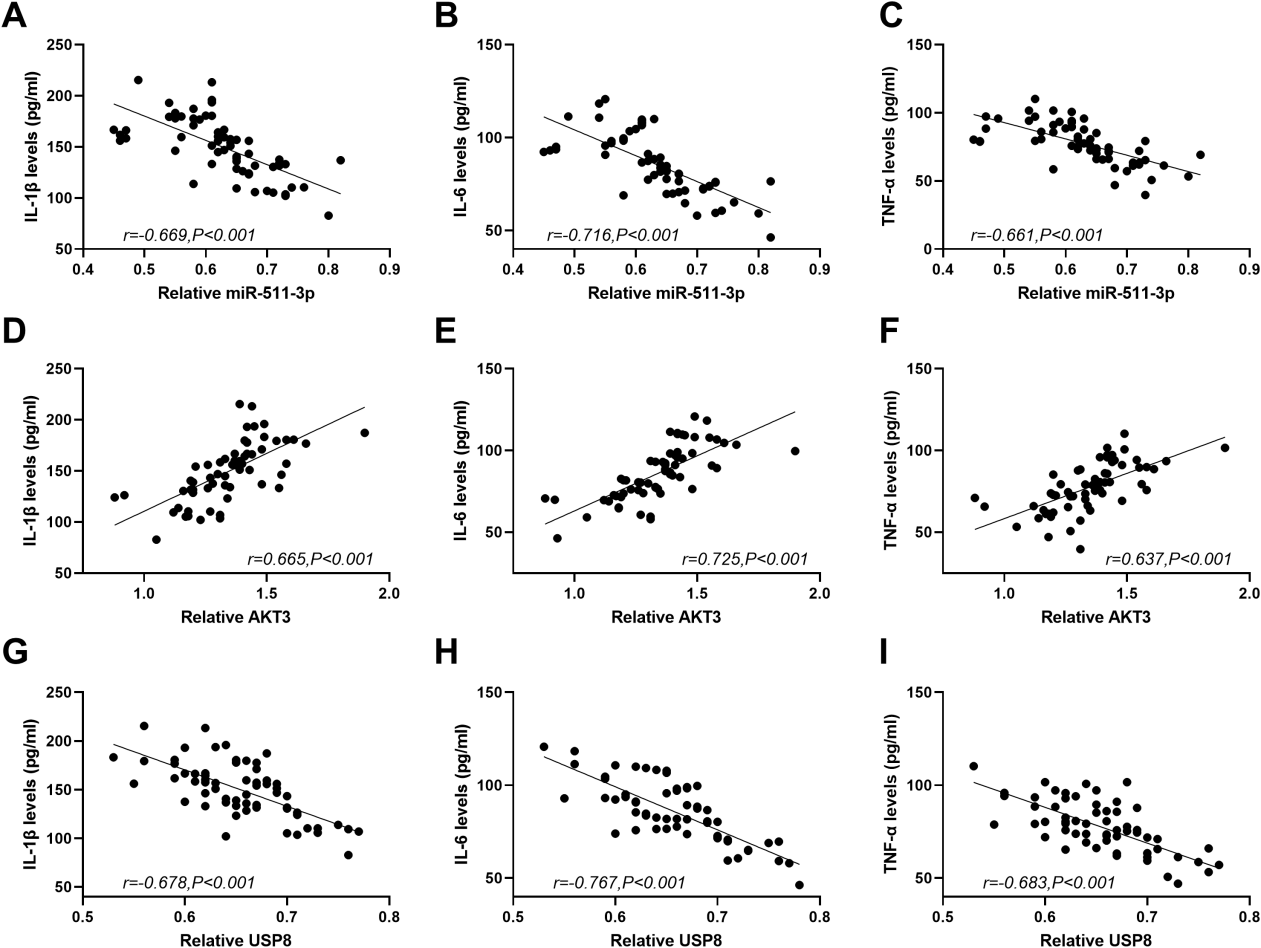
The correlation between miR-511-3p, AKT3, USP8 and inflammatory factors in patients with PSCI. **(A–C)**. The relative expression level of miR-511-3p was significantly negatively correlated with the levels of IL-1β, IL-6, and TNF-α. **(D–F)**. The relative expression level of AKT3 was significantly positively correlated with the levels of IL-1β, IL-6, and TNF-α. **(G–I)**. The relative expression level of USP8 was significantly negatively correlated with the levels of inflammatory factors.

Using the Pearson correlation coefficient analysis method, the correlations among miR-511-3p, AKT3, USP8, and inflammatory cytokines were analyzed. The results were shown in **Figure 5**. The results indicated that the expression level of miR-511-3p in the serum of PSCI patients was negatively correlated (**Figures 5A–C**) with IL-1β (r= -0.669, *P* < 0.001), IL-6 (r= -0.716, *P* < 0.001), and TNF-α (r= -0.661, *P* < 0.001). In contrast, the expression level of AKT3 was positively correlated with IL-1β (r= 0.665, *P* < 0.001), IL-6 (r= 0.725, *P* < 0.001), and TNF-α (r= 0.637, *P* < 0.001). The expression level of USP8 was negatively correlated with IL-1β (r= -0.678, *P* < 0.001), IL-6 (r= -0.767, *P* < 0.001), and TNF-α (r= -0.683, *P* < 0.001).

### Logistic regression analysis of PSCI risk factors

The results of univariate Logistic regression analysis (**Table 3**) showed that low expression of miR-511-3p and USP8, and high expression of AKT3 were associated with the risk of PSCI (*P* < 0.05). After adjusting for age, gender, smoking status, drinking status, diabetes and hypertension in the multivariate Logistic regression analysis (**Table 4**), low expression of miR-511-3p and USP8 remained independent risk factors for PSCI (*P* < 0.001), while the influence of AKT3 was no longer significant. Age, gender, smoking, drinking, diabetes, and hypertension were not significantly associated with the risk of PSCI in both univariate and multivariate analyses.

**Table 3:** Logistic univariate regression analysis for evaluating the risk factors of PSCI.

	OR	95% CI	P-value
Age	1.005	0.487-2.071	0.990
Gender	1.102	0.532-2.281	0.794
Smoking	1.591	0.760-3.331	0.218
Drinking	0.843	0.406-1.750	0.647
Diabetes	1.265	0.610-2.622	0.528
hypertension	0.738	0.354-1.540	0.419
miR-511-3p	0.037	0.014-0.099	<0.001
AKT3	0.434	0.207-0.910	0.027
USP8	0.041	0.015-0.108	<0.001

**Table 4:** Logistic multivariate regression analysis for evaluating the risk factors of PSCI.

	OR	95% CI	P-value
Age	2.029	0.492-8.369	0.328
Gender	2.023	0.498-8.215	0.324
Smoking	1.480	0.367-5.976	0.582
Drinking	1.227	0.293-5.126	0.780
Diabetes	1.106	0.281-4.351	0.885
hypertension	0.329	0.075-1.454	0.143
miR-511-3p	0.028	0.006-0.125	<0.001
AKT3	0.448	0.115-1.740	0.246
USP8	0.028	0.006-0.134	<0.001

### Accuracy evaluation and validation of the prediction model for PSCI

**Figure 6:**
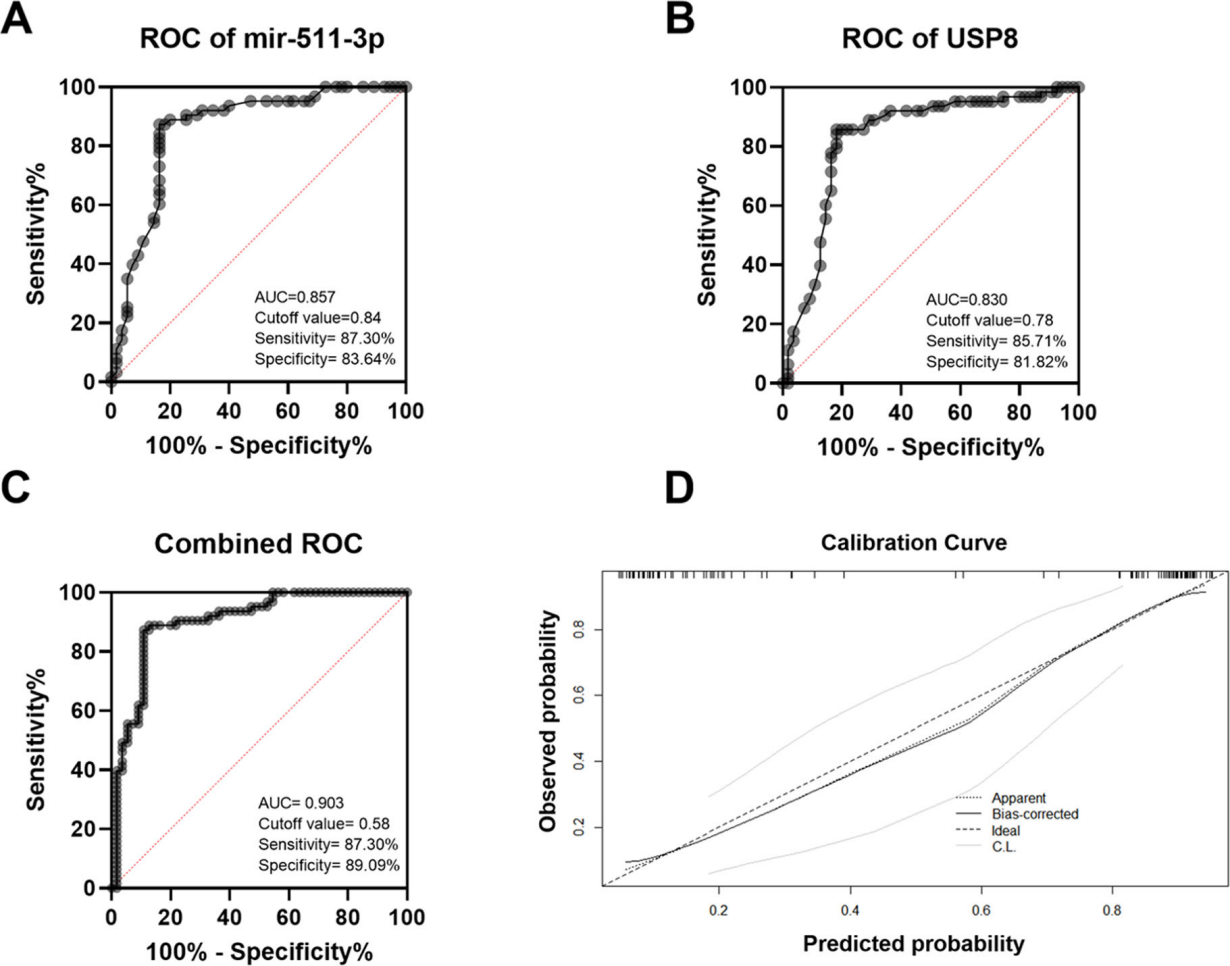
Diagnostic performance of miR-511-3p, USP8, and their combined model for PSCI **(A)** ROC curve of miR-511-3p (AUC = 0.857). **(B)** ROC curve of USP8 (AUC = 0.830). **(C)** Combined ROC curve of miR-511-3p and USP8 (AUC = 0.903). **(D)** Calibration curve of the combined model, showing agreement between predicted and observed probabilities.

ROC curve analysis was performed to evaluate the diagnostic performance of miR-511-3p, USP8, and their combined model for predicting PSCI. The AUC for miR-511-3p was 0.857 (**Figure 6A**), while the AUC for USP8 was 0.830 (**Figure 6B**), indicating that both biomarkers exhibited good diagnostic performance. Notably, the combined model achieved a higher AUC of 0.903 (**Figure 6C**), demonstrating superior predictive accuracy compared to either biomarker alone. The calibration curve (**Figure 6D**) demonstrated good agreement between the predicted probabilities and the observed outcomes for the combined model. The Hosmer–Lemeshow test yielded a χ² value of 13.56 with a P value of 0.094, indicating satisfactory calibration of the model. After 1,000 bootstrap resamples, the combined model showed a prediction accuracy of 88.2% (95% CI: 82.2%–93.2%), suggesting good discriminative ability, calibration, and internal stability.

## Discussion

Neuroinflammation is one of the important pathological mechanisms of brain damage after stroke, involving the activation of cytokines, chemokines and immune cells (21). The pathological damage after a stroke is not limited to the acute neuronal necrosis caused by ischemia/hemorrhage; the subsequent continuous neuroinflammatory response is a key link in exacerbating brain damage and promoting the occurrence of PSCI (22). Although studies have confirmed that neuroinflammation is closely related to PSCI, the molecular mechanism of how neuroinflammatory signals are precisely transmitted to the cognitive regulatory pathways remains unclear. Therefore, further exploration of the pathogenesis of neuroinflammation after stroke and the connection bridge with the occurrence of PSCI has significant social significance.

In this study, we demonstrated the regulatory effect of miR-511-3p/AKT3/USP8 on the inflammatory response in a neural inflammation cell model. Additionally, in patients, it was confirmed that the expressions of miR-511-3p, AKT3, and USP8 were associated with the levels of inflammatory factors. Furthermore, our analysis revealed that low expression of miR-511-3p and USP8 were an independent risk factor for PSCI. ROC analysis confirmed that miR-511-3p and USP8 had good diagnostic value for PSCI (AUC: 0.857 and 0.830). The combined model achieved a higher AUC of 0.903, showing superior predictive accuracy. Calibration curve and Hosmer–Lemeshow test verified satisfactory model calibration, and bootstrap validation demonstrated high accuracy (88.2%, 95% CI: 82.2%–93.2%) with good internal stability. These findings suggest that the combination of miR-511-3p and USP8 could improve the diagnostic efficiency for PSCI.

miRNAs are a class of endogenous non-coding single-stranded RNA molecules, usually 19 to 25 nucleotides in length, which play an important role in regulating gene expression (23). miRNAs show a high degree of conservation in different species, and their sequences and functions remain stable during evolution (24). It has been found that microRNAs interact with the 3’-UTR of target mRNAs. Bioinformatics target prediction is usually the first step to determine the function of microRNA and find its target genes (25). The principle of luciferase reporter gene detection is to fuse the promoter region of the target gene with the luciferase gene to construct a reporter gene plasmid and verify the interaction between the miRNA and its target gene (26). In this study, through bioinformatics prediction by the starBase and a dual-luciferase reporter gene technology, it was confirmed that miR-511-3p could target and bind to the upstream promoter region of AKT3. Furthermore, in BV2 cells, the overexpression of miR-511-3p led to inhibited AKT3, and the inhibition of miR-511-3p led to promoted AKT3. These results indicated that AKT3 was a direct target gene of miR-511-3p, and might mediate the function of miR-511-3p.

miR-511-3p may reduce neuroinflammation by regulating the expression of inflammatory factors or affecting the response of neurons and glial cells (27). AKT activation can reduce neuronal apoptosis, inhibit the expression of inflammatory factors, and improve neurological function (28). And miR-511-3p activates the AKT signaling pathway by targeting PTEN, which further regulates the expression of inflammatory factors (9). In a model of sepsis-associated encephalopathy, USP8 can improve cognitive and motor dysfunction by inhibiting the activation of microglia (29). Meanwhile, the knockout of USP8 inhibits the activation of AKT signaling pathway, thereby reducing cell apoptosis (30). The interaction among the three may have important implications for the regulation of neuroinflammation. Hence this study constructed a stroke-related inflammatory cell model by OGD/R stimulation in BV2 cells. Compared with BV2 cells, the expression levels of miR-511-3p and USP8 in BV2 OGD/R cells were significantly decreased, and the expression level of AKT3 was significantly increased. Overexpression of miR-511-3p significantly increased miR-511-3p levels and was not inhibited by pcDNA3.1-AKT3 (**Figure 2D**). Overexpression of miR-511-3p significantly attenuated AKT3 expression, which was reversed by pcDNA3.1-AKT3 (**Figure 2E**). miR-511-3p mimic enhanced USP8 expression, but the enhancement was inhibited by pcDNA3.1-AKT3 (**Figure 2F**). Overall, miR-511-3p could regulate AKT3/USP8 expression and AKT3 could regulate USP8 expression in the OGD/R-induced neuroinflammation model. Based on the above discovery, we speculated that miR-511-3p, AKT3 and USP8 may constitute a regulatory cascade involved in the progression of neuroinflammation.

To explore the role of miR-511-3p/AKT3/USP8 signaling axis in neuroinflammation, the expression of IL-1β, IL-6 and TNF-α was examined in BV2 cell model. IL-1β, IL-6, and TNF-α are important proinflammatory cytokines in neuroinflammation. TNF-α can promote the activation of the inflammasome and amplify the inflammatory response through the NF-κB signaling pathway (31). IL-1β is involved in neuronal apoptosis and inflammasome activation (32), and IL-6 exacerbates neurodegenerative diseases by promoting inflammatory responses and neuronal damage (33). According to upregulating the expression of miR-511-3p or USP8, the concentration of IL-1β, IL-6, and TNF-α were significantly inhibited. Reversely, the levels of inflammatory cytokines were promoted by the overexpression of AKT3. The rescued inflammatory responses by AKT3 overexpression in the cells with miR-511-3p overexpression indicated that AKT3/USP8 might mediate the regulatory effects of miR-511-3p on inflammation in neuroinflammation model cells.

To further confirm the role of miR-511-3p/AKT3/USP8 in PSCI, we recruited 118 stroke patients. The expression levels of miR-511-3p and USP8 in the serum of PSCI patients were decreased, while the expression of AKT3 was increased. Moreover, all three factors were associated with the MoCA score and the levels of inflammatory factors. The low expression of miR-511-3p may weaken the inhibition of AKT3, leading to an increase in AKT3 expression, and subsequently promoting the development of PSCI through inflammatory-related pathways. This is similar to the research results in other neurological diseases where serum markers are associated with the disease, such as abnormal expression of certain miRNAs and inflammatory factors in the serum of stroke patients being related to the severity of the disease (34). Finally, the Logistic regression analysis indicated that the low expression of miR-511-3p and USP8 were independent risk factors for PSCI. This result validated the conclusion of the molecular mechanism study from the perspective of clinical epidemiology, demonstrating that miR-511-3p and USP8 not only participate in the PSCI-related processes at the cellular and molecular levels, but are also closely related to the risk of PSCI in clinical phenotypes. This provides potential biomarkers for the risk assessment and early diagnosis of PSCI. However, AKT3 showed no significant association in the multivariate analysis. This might be due to its more significant role in the intermediate links of the molecular pathways, being more regulated by other factors, or being affected by sample size and other factors, which reduced the statistical power.

In conclusion, this study demonstrates that miR-511-3p regulates USP8 by targeting AKT3, thereby influencing the neuroinflammatory response and possibly participating in the occurrence and development mechanism of PSCI. Moreover, miR-511-3p and USP8 are expected to become biomarkers and therapeutic targets for PSCI.

## Data Availability

The original contributions presented in the study are included in the article/supplementary material. Further inquiries can be directed to the corresponding author.
